# Probable Sporadic Creutzfeldt-Jakob Disease Presenting as Rapidly Progressive Dementia: A Case Report From Georgia

**DOI:** 10.7759/cureus.114855

**Published:** 2026-08-20

**Authors:** Nikoloz Kvachadze, Giorgi Kotia, Mariam Muradashvili, Shota Mrelashvili, Nikoloz Basilaia, Luka Zambakhidze

**Affiliations:** 1 Intensive Care, Simon Khechinashvili University Clinic, Tbilisi, GEO; 2 Medicine, Caucasus International University, Tbilisi, GEO; 3 Emergency Medicine, Tbilisi State Medical University, Tbilisi, GEO; 4 Biomedical Sciences, Georgian-American University, Tbilisi, GEO; 5 Medicine, Tbilisi State Medical University, Tbilisi, GEO

**Keywords:** acute hypoxic respiratory failure, csf 14-3-3 protein, gait ataxia, human prion disease, rapidly progressive dementia, sporadic creutzfeldt-jakob disease (scjd)

## Abstract

Creutzfeldt-Jakob disease (CJD) is a rare, fatal prion disorder that commonly presents as rapidly progressive dementia and may initially mimic autoimmune, paraneoplastic, infectious, or metabolic encephalopathies. We report a case of probable sporadic CJD in a 53-year-old man from Georgia who developed approximately one month of progressive gait difficulty, dysarthria, behavioral changes, and memory impairment. Neurological examination revealed hypophonic dysarthric speech, horizontal nystagmus, mild-to-moderate tetraparesis, dystonic hyperkinesia, distal sensory impairment, and severe gait ataxia with astasia-abasia. Brain computed tomography showed no acute intracranial lesion, while magnetic resonance imaging demonstrated restricted diffusion involving the head and body of the left caudate nucleus. Electroencephalography (EEG) demonstrated nonspecific findings. Testing for a defined set of autoimmune and paraneoplastic antibodies was negative, and positron emission tomography showed no evidence of malignancy. Electroneuromyography demonstrated mixed demyelinating and axonal peripheral nerve involvement. Cerebrospinal fluid (CSF) analysis showed no pleocytosis, a mildly elevated protein level, and a positive CSF 14-3-3 protein result, supporting the diagnosis of probable sporadic CJD. Real-time quaking-induced conversion (RT-QuIC) testing was not performed because it was unavailable in the treating clinical setting.

Empirical high-dose methylprednisolone produced no meaningful neurological improvement. The patient subsequently progressed to a bedbound, nonverbal state with severe dependence and recurrent respiratory complications. Despite prolonged intensive care, mechanical ventilation, tracheostomy, and broad-spectrum antimicrobial therapy, he died approximately seven months after symptom onset from pneumonia complicated by acute respiratory failure and shock, culminating in cardiac arrest. This case highlights the diagnostic challenges of rapidly progressive dementia and the importance of considering sporadic CJD when cognitive decline is accompanied by cerebellar, pyramidal, extrapyramidal, and peripheral neurological features.

## Introduction

Creutzfeldt-Jakob disease (CJD) is a rare, rapidly progressive, and invariably fatal prion disorder within the group of transmissible spongiform encephalopathies [1,2]. It results from misfolding of normal cellular prion protein into an abnormal pathogenic isoform, causing self-propagating protein aggregation, neuronal injury, gliosis, and characteristic spongiform change in the central nervous system [1]. Although uncommon, CJD is clinically important because it often presents as rapidly progressive dementia, a neurological emergency requiring prompt evaluation for potentially reversible causes [3,4].

CJD is classified as sporadic, genetic, iatrogenic, or variant. Sporadic CJD is the most common form and usually occurs without a recognized transmission pattern, family history, or identifiable exposure [2]. Before considering prion disease, clinicians must exclude potentially treatable causes of rapidly progressive dementia, including autoimmune encephalitis, paraneoplastic syndromes, infections, metabolic disorders, vascular disease, and malignancy-associated neurological syndromes [3-5]. As many of these conditions may initially resemble CJD, diagnosis is often delayed until alternative explanations are systematically ruled out [3-5].

Modern neuroimaging plays an important role in supporting the diagnosis of sporadic CJD (sCJD), often demonstrating characteristic cortical or deep gray matter changes [6,7]. However, these findings may be absent or nonspecific early in the disease, making clinical suspicion more difficult [6,7]. Cerebrospinal fluid (CSF) biomarkers, including 14-3-3 protein, provide additional support but are not disease-specific and must be interpreted within the overall clinical context [8].

This case captures the diagnostic tension of rapidly progressive dementia between probable sporadic CJD and competing diagnostic possibilities, particularly when early clinical features do not immediately establish a prion disease [3-5,9]. The diagnosis of probable sporadic CJD relies on the convergence of a compatible clinical phenotype and supportive investigations rather than on any single biomarker. In this case, the coexistence of atypical central and peripheral neurological features broadened the initial differential diagnosis. The case further highlights the transition from diagnostic uncertainty to advanced neurodegenerative dependence, impaired airway protection, respiratory infection, and fatal outcome [2].

## Case presentation

A 53-year-old man was evaluated for a rapidly progressive neurological syndrome that had evolved over approximately one month. His illness began with gradually worsening gait difficulty, speech changes, behavioral alterations, and memory impairment. Shortly before hospital admission, he developed involuntary movements of the left upper extremity, followed by increasing confusion, inappropriate behavior, and disorientation.

On Day 1, he presented to the emergency department with progressive weakness in all four limbs and inability to walk independently. Earlier neurological assessment had documented memory decline and balance disturbance. HIV and syphilis serology were nonreactive, and autoimmune antineuronal antibodies, including anti-LGI1, anti-CASPR2, and anti-NMDA (N-methyl-D-aspartate) receptor antibodies, as well as the paraneoplastic onconeural antibody anti-Hu (ANNA-1), were not detected; the findings and their clinical interpretation are detailed in Table 1. At admission, the preliminary differential diagnosis included unspecified encephalitis, myelitis, and encephalomyelitis, with autoimmune and paraneoplastic etiologies considered.

**Table 1:** Serological findings and their clinical interpretation NMDA: N-methyl-D-aspartate.

Serological test	Patient result	Interpretation	Clinical significance
Human immunodeficiency virus screening	Nonreactive/negative	No serological evidence of HIV infection	Helps exclude HIV-related neurological disease as a potentially treatable cause of rapidly progressive neurological decline.
Syphilis serology	Nonreactive/negative	No serological evidence of syphilis	Helps exclude neurosyphilis as a potentially reversible cause of rapidly progressive dementia.
Autoimmune antineuronal and paraneoplastic onconeural antibodies (anti-LGI1, anti-CASPR2, anti-NMDA receptor, and anti-Hu (ANNA-1))	Not detected	None of the tested circulating autoimmune antineuronal or paraneoplastic onconeural antibodies were detected	Reduces support for the tested autoimmune encephalitis and paraneoplastic neurological syndromes, although a negative result does not exclude seronegative autoimmune encephalitis.

Neurological examination revealed an asthenic, hypodynamic patient with hypophonic dysarthric speech. Horizontal nystagmus was observed during bilateral abduction, with possible isolated opsoclonus. Motor examination demonstrated mild-to-moderate tetraparesis, tetra-ataxia, brisk tendon reflexes without pathological reflexes, and dystonic hyperkinesia of the left upper extremity. Sensory examination showed distal superficial hypoesthesia in a polyneuropathic distribution. Gait was severely ataxic, with astasia-abasia requiring bilateral assistance, and spontaneous fasciculations were observed in the shoulder-girdle region. The coexistence of brisk reflexes and tetraparesis with fasciculations and sensory abnormalities suggested a mixed central and peripheral neurological phenotype, broadening the differential diagnosis.

**Figure 1:**
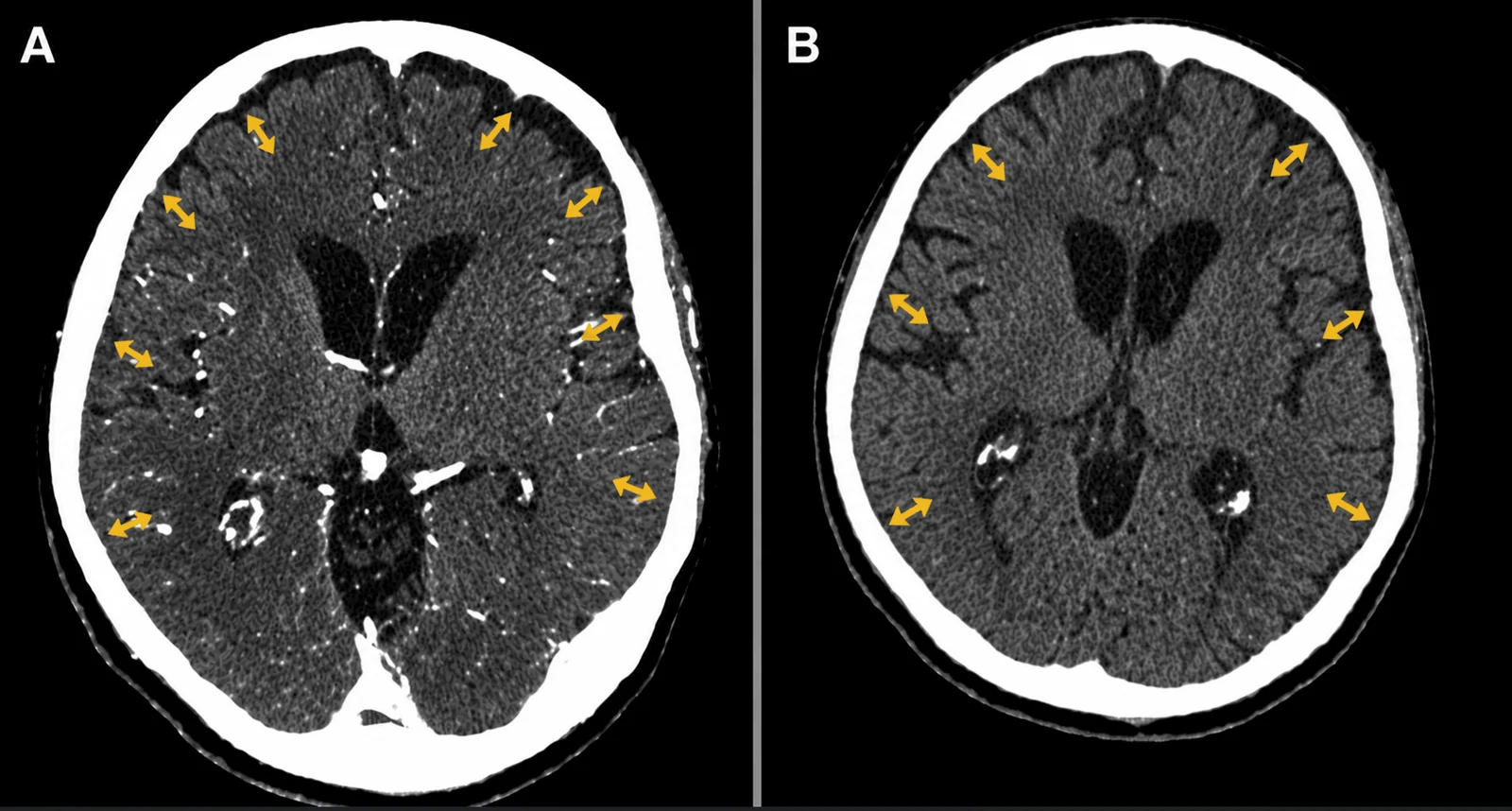
Initial contrast-enhanced brain CT demonstrating diffuse cerebral cortical atrophy. In panels A and B, the orange arrows indicate prominence and widening of the cortical sulci, consistent with generalized cortical volume loss. No acute focal intracranial lesion is identified.

Initial contrast-enhanced brain computed tomography showed no acute focal intracranial lesion or abnormal focal enhancement but demonstrated diffuse cortical atrophic changes, including prominence and widening of the cortical sulci, as illustrated in Figure 1. These findings did not identify a single structural explanation for the patient’s rapidly progressive cognitive and neurological decline. 

**Figure 2:**
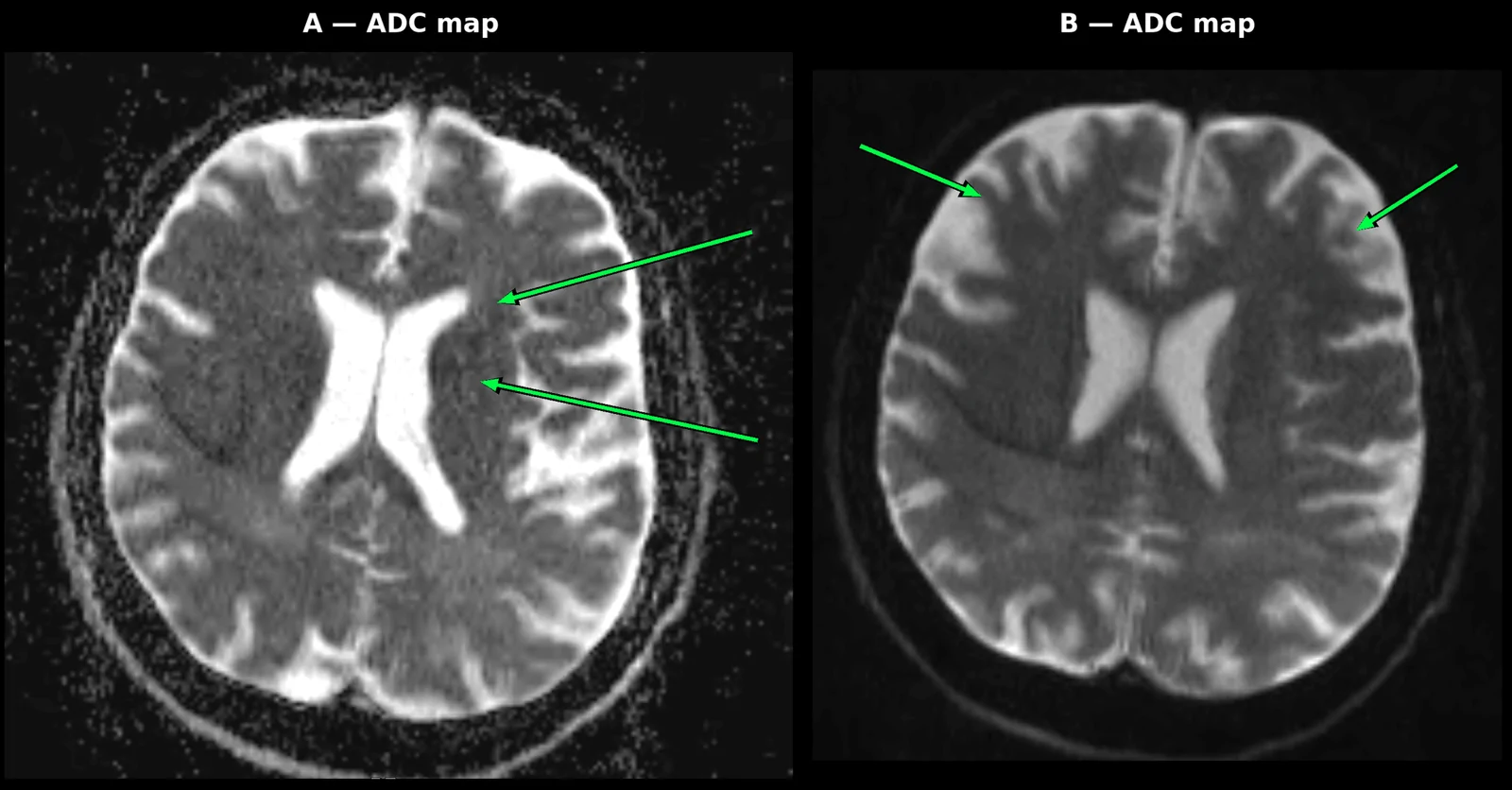
Axial diffusion MRI images (ADC maps) with green arrows indicating the described abnormalities. (A) Reduced signal in the head and body of the left caudate nucleus, consistent with restricted diffusion, with mildly increased volume compared with the contralateral caudate nucleus. (B) Moderate dilatation of the convexity subarachnoid spaces. ADC: Apparent diffusion coefficient.

Subsequent brain MRI demonstrated restricted diffusion involving the head and body of the left caudate nucleus, with mild asymmetric enlargement, along with moderate dilatation of the convexity subarachnoid spaces, as demonstrated in Figure 2. In the context of rapidly progressive cognitive deterioration, cerebellar dysfunction, abnormal movements, motor impairment, and sensory abnormalities, the combined CT and MRI findings supported the presence of diffuse cerebral involvement while reinforcing the need for a broader diagnostic evaluation for prion disease and potentially treatable mimics [3,4,6,7].

Electroencephalography (EEG) performed during the initial diagnostic evaluation demonstrated nonspecific findings without a characteristic pattern suggestive of CJD. The presentation was rapidly progressive and polymorphic; the diagnostic workup was directed toward both treatable mimics and prion disease [3-5,9,10]. Autoimmune and paraneoplastic encephalitis were strongly considered. Positron emission tomography showed no evidence of malignancy. However, the rapid clinical decline and multifocal neurological involvement raised concern for CJD [3-5,9]. CSF analysis demonstrated no pleocytosis, mildly elevated protein, and positive CSF 14-3-3 protein testing, as presented in Table 2.

**Table 2:** Cerebrospinal fluid analysis and CSF 14-3-3 protein testing The positive 14-3-3 result was interpreted in the context of no pleocytosis, mildly elevated protein, rapidly progressive neurological decline, and exclusion of competing diagnoses. SCJD: Sporadic Creutzfeld–Jakob disease.

CSF parameters	Result	Reference interval	Clinical interpretation
Macroscopic appearance	Clear and colorless	Clear and colorless	Normal
Nucleated cell count	2 cells/µL	<5 cells/µL	No pleocytosis
Total protein	0.455 g/L (45.5 mg/dL)	0.22–0.33 g/L (22–33 mg/dL)	Mildly elevated
Glucose	3.33 mmol/L	2.2–3.9 mmol/L	Within reference range
CSF 14-3-3 protein	Positive	Negative/not detected	Supportive of probable sCJD in the appropriate clinical context; not disease-specific

This result was interpreted as supportive rather than definitive, given that 14-3-3 protein reflects neuronal injury and is not specific for CJD [8]. CSF real-time quaking-induced conversion (RT-QuIC) testing was not performed because the assay was unavailable in the treating clinical setting during the patient's diagnostic evaluation. The patient received empiric high-dose methylprednisolone without meaningful neurological improvement. In the absence of a more convincing alternative diagnosis, the overall clinical course supported probable sporadic CJD [9].

**Figure 3:**
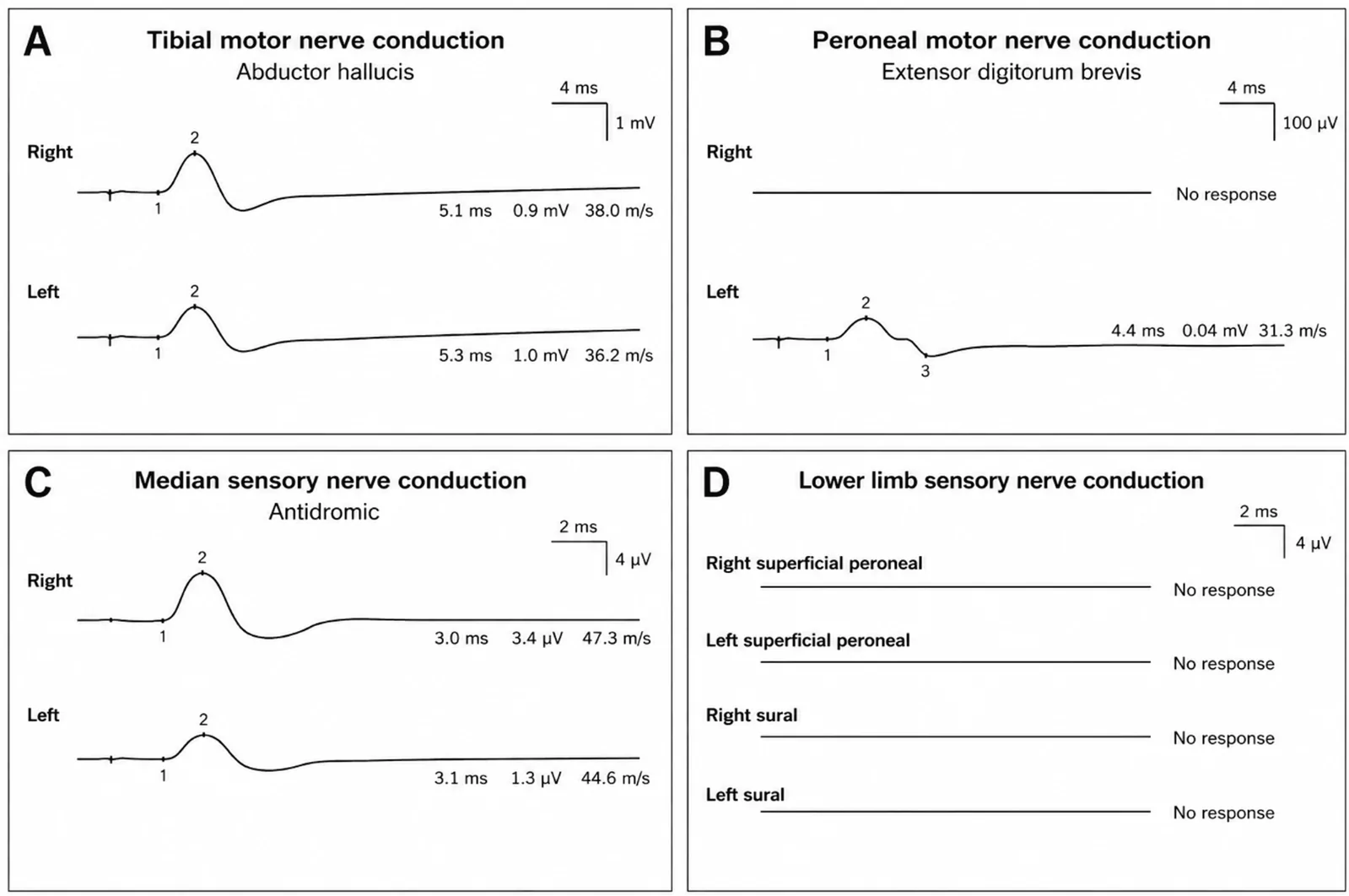
Schematic representation of the electroneuromyography findings (A) Bilateral tibial motor nerve conduction studies demonstrate markedly reduced compound muscle action potential amplitudes with mildly decreased conduction velocities. (B) No reliable motor response was recorded from the right peroneal nerve, while the left peroneal nerve showed a severely reduced motor amplitude and slowed conduction. (C) Bilateral median sensory nerve conduction studies demonstrate markedly reduced sensory nerve action potential amplitudes with mildly decreased conduction velocities. (D) No reliable sensory responses were recorded from the bilateral superficial peroneal or sural nerves. Overall, the findings were consistent with severe sensorimotor axonal polyneuropathy with additional demyelinating features.

Electroneuromyography was performed because of prominent limb weakness, sensory abnormalities, and fasciculations. The study demonstrated mixed peripheral nerve involvement with demyelinating and axonal features, including conduction block in both tibial nerves, motor axonopathy involving the left peroneal nerve, and reduced sensory potential amplitudes in the superficial peroneal and sural nerves, as shown in Figure 3.

Over the following months, the patient’s illness progressed from diagnostic uncertainty to severe neurological dependence. He became bedbound, lost verbal communication, required continuous care, and developed episodes suggestive of impaired swallowing and poor airway protection. During a later hospitalization for fever, cough, sputum production, and generalized weakness, inflammatory markers were elevated, although respiratory and hemodynamic parameters were initially relatively stable, and a clear pneumonic focus was not established radiographically. He was treated with supportive care, oxygen therapy as needed, intravenous fluids, inhaled bronchodilator and corticosteroid therapy, anticoagulant prophylaxis, and empiric antimicrobial therapy. After discharge, outpatient management remained supportive, including continued neurological follow-up.

After discharge, outpatient care remained supportive and symptom-directed, with continued neurological follow-up, general nursing care, feeding and aspiration precautions, and antihypertensive therapy as needed. No disease-modifying treatment for suspected CJD was available, and management focused on comfort, prevention of complications, and monitoring for respiratory deterioration [11].

His subsequent admission marked a transition from advanced neurological dependence to life-threatening respiratory failure. He returned with fever, sweating, cough, difficult sputum clearance, severe weakness, and worsening feeding and swallowing. A repeat 3-Tesla brain MRI had been planned for further diagnostic clarification but could not be performed because of his severe general condition. 

On Day 144, upon readmission, he was febrile, tachycardic, tachypneic, and markedly hypoxemic. Neurologically, he was somnolent, nonverbal, unable to follow commands, and exhibited no meaningful limb movement. Laboratory testing demonstrated neutrophilic leukocytosis, a marked inflammatory response, electrolyte disturbances, renal dysfunction, and coagulation abnormalities, as summarized in Table 3.

**Table 3:** Laboratory findings during the later admission

Test name	Result	Unit	Reference interval
White blood cell count (WBC)	18.34	10^9^/L	4.2–10
Neutrophils, absolute count	15.70	10^9^/L	2.1–7.2
Neutrophils	85.59	%	50–72
Band neutrophils	10.00	%	1–6
Segmented neutrophils	72.00	%	40–65
C-reactive protein (CRP)	291.72	mg/L	<5
Creatinine	143.00	µmol/L	Adult male: 59–104
Sodium (Na+)	154.00	mmol/L	Adult: 136–145
Potassium (K+)	3.90	mmol/L	Adult: 3.5–5.1
Chloride (Cl−)	115.00	mmol/L	Adult: 98–107
Prothrombin time (PT)	16.30	s	9.5–13.0
Prothrombin index (PI)	57.00	%	70.0–115.0
International normalized ratio (INR)	1.50		0.9–1.2
Hemoglobin (HGB)	14.91	g/dL	Adult male: 13.5–17.5
Platelet count (PLT)	396.00	10^9^/L	150–400

Chest imaging obtained at the beginning of this later admission demonstrated predominantly right lower-lung infiltrative and consolidative changes, with bilateral involvement and retained bronchial secretions. The overall picture reflected advanced neurodegenerative dependence complicated by impaired airway protection, secretion retention, and severe respiratory infection [11].

**Figure 4:**
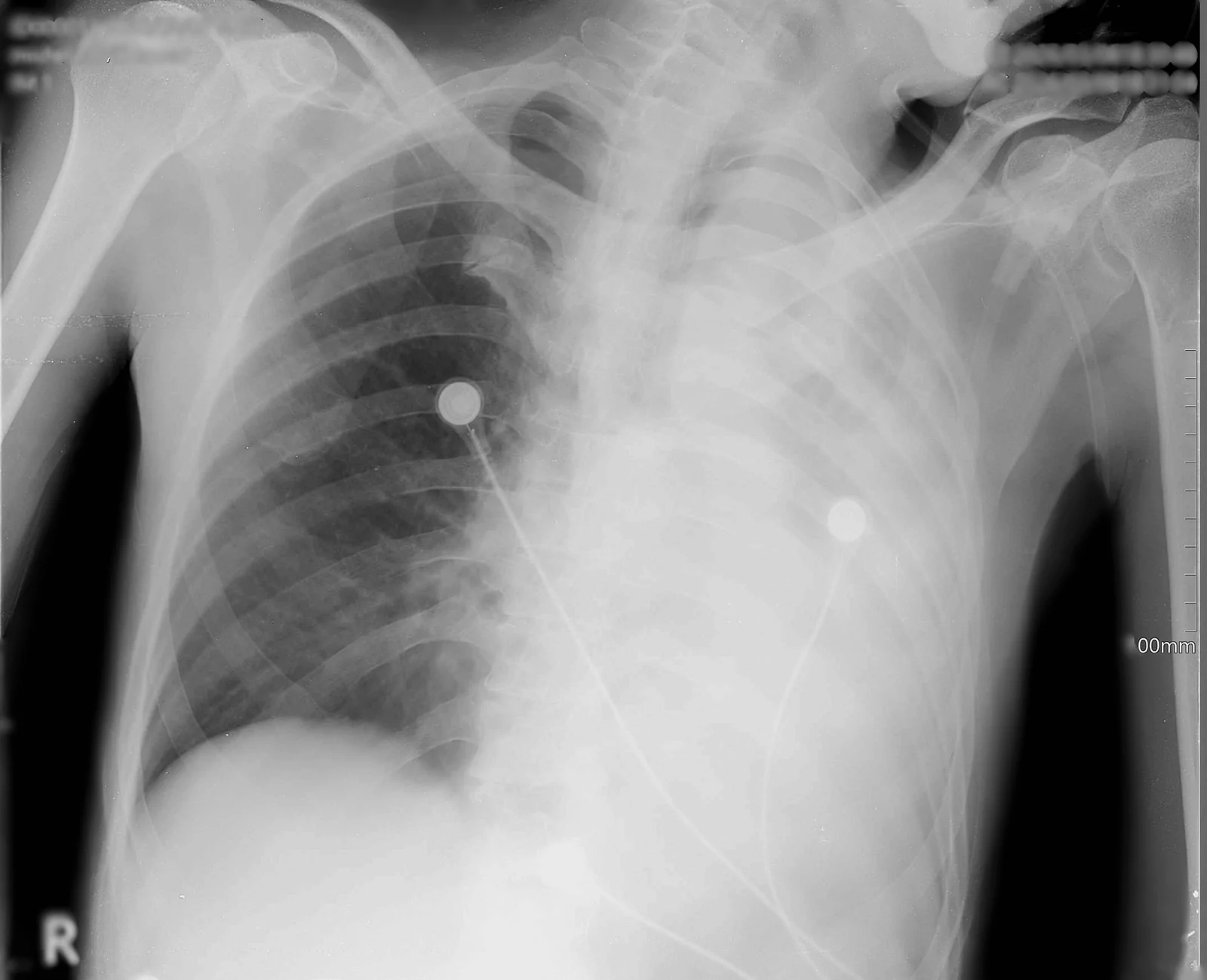
Portable anteroposterior chest radiograph obtained during the terminal respiratory course demonstrating near-complete opacification of the left hemithorax due to extensive left-sided air-space consolidation with associated pleural effusion, consistent with severe pneumonia

Because of worsening respiratory failure and ineffective secretion clearance, he required repeated suctioning, bronchoscopy with bronchial lavage, oxygen therapy, noninvasive continuous positive airway pressure (CPAP) support, broad-spectrum antimicrobial treatment, nasogastric feeding, urinary catheterization, and later mechanical ventilation through a tracheostomy tube. A portable chest radiograph obtained later, during the terminal respiratory course, demonstrated near-complete opacification of the left hemithorax, as shown in Figure 4.

Despite prolonged supportive and critical care management, the patient continued to deteriorate and died on Day 183, approximately seven months after symptom onset. The final documented diagnosis included pneumonia complicated by acute respiratory failure, shock, pleural effusion, anemia, and cardiac arrest, with probable CJD recorded among the associated neurological diagnoses. In the absence of neuropathological confirmation, the case was classified as probable sporadic CJD [9,11].

## Discussion

The central dilemma is to pursue reversible causes with urgency while recognizing the more ominous signal of a disease that often reveals itself not through a single defining test but through the force of its relentless clinical progression [3-5,9]. Sporadic CJD occupies this difficult diagnostic space [2,9]. It is a fatal prion disorder caused by conversion of normal cellular prion protein into a misfolded pathogenic form, leading to self-propagating protein aggregation, neuronal loss, gliosis, and spongiform neurodegeneration [1]. Its clinical expression is heterogeneous and may include cognitive decline, behavioral change, cerebellar dysfunction, pyramidal or extrapyramidal signs, abnormal movements, visual symptoms, akinetic mutism, and rapid functional collapse [1,2,9].

Modern diagnostic standards increasingly emphasize convergence rather than reliance on a single test [6-10]. RT-QuIC has become the most important recent diagnostic advance because it detects prion seeding activity with high specificity, but it remains unavailable in many clinical settings [9,10]. This limitation applied to the present case, as RT-QuIC testing was not available in the treating clinical setting and therefore could not be performed. MRI, particularly diffusion-weighted imaging (DWI) with corresponding apparent diffusion coefficient (ADC) maps and fluid-attenuated inversion recovery (FLAIR) sequences, can provide strong supportive evidence through cortical or deep gray matter abnormalities, yet imaging may be nonspecific, incomplete, or impossible to repeat when patients deteriorate [6,7,9]. CSF 14-3-3 protein remains useful, but it is best understood as a marker of rapid neuronal injury rather than a disease-specific marker of CJD [8,9]. Therefore, the diagnosis is strongest when biomarker evidence aligns with a compatible phenotype, low-inflammatory CSF findings, exclusion of treatable alternatives, and relentless progression [3-10].

The instructive value of this case lies in the interval between the first neurological symptoms and the point at which probable sporadic CJD became the most coherent explanation [3-5,9]. Early in the course, the patient did not present with a single defining feature but with a changing constellation of cognitive decline, behavioral disturbance, dysarthria, gait failure, abnormal movements, motor impairment, and sensory findings. This type of presentation is precisely where rapidly progressive dementia becomes diagnostically demanding: the clinician must keep prion disease in view while still actively excluding disorders that may be treatable [3-5].

The initial diagnosis of unspecified encephalitis, myelitis, and encephalomyelitis was therefore clinically reasonable. The early picture overlapped with inflammatory, autoimmune, paraneoplastic, infectious, and metabolic causes of rapidly progressive neurological decline [3-5]. Over time, however, several findings increasingly supported sCJD: unrevealing routine infectious and metabolic testing, absent serum antineuronal antibodies, lack of meaningful response to high-dose corticosteroids, and continued neurological deterioration [9].

The final course shows how the fatality of CJD may become clinically visible through secondary complications of neurological collapse [11]. As the patient became bedbound and nonverbal, impaired swallowing, poor secretion clearance, and loss of airway protection became central problems. The terminal admission demonstrated respiratory infection with hypoxemia, leukocytosis, elevated inflammatory markers, retained bronchial secretions, pneumonia, and acute respiratory failure. In this way, the outcome reflected both progressive brain disease and the predictable consequences of advanced neurodegenerative dependence: aspiration risk, infection, ventilatory failure, intensive care requirement, tracheostomy, and death [11].

## Conclusions

Probable sporadic CJD should remain in the differential diagnosis when rapidly progressive dementia is accompanied by cerebellar dysfunction, dysarthria, abnormal movements, pyramidal or extrapyramidal signs, and rapid loss of independence. This case emphasizes that the diagnosis often develops through careful longitudinal assessment, supportive biomarker evidence, and systematic exclusion of treatable mimics. CSF 14-3-3 protein may support the diagnosis but only when interpreted within the full clinical context. In this case, the diagnosis remained probable rather than definite, based on the compatible clinical course, MRI findings, positive CSF 14-3-3 result, and exclusion of important alternative diagnoses. However, diagnostic uncertainty persisted because RT-QuIC testing was unavailable and neuropathological confirmation was not obtained. The case also highlights the need to anticipate the later consequences of advanced neurodegenerative disease, including impaired swallowing, secretion retention, pneumonia, acute respiratory failure, and the need for early family-centered supportive planning.
